# Virulence Phenotypes Differentiate Persistent vs. Resolving Isolates of Human *Staphylococcus aureus* Bacteremia

**DOI:** 10.3390/antibiotics15040332

**Published:** 2026-03-25

**Authors:** Liana C. Chan, Hong K. Lee, Ling Wang, Huiyuan Wang, Scott G. Filler, Alexandra Ciranna, Wessam Abdelhady, Yan Q. Xiong, Liang Li, Rachelle A. Gonzales, Felicia Ruffin, Vance G. Fowler, Arnold S. Bayer, Richard A. Proctor, Michael R. Yeaman

**Affiliations:** 1 Harbor-UCLA Medical Center, Division of Molecular Medicine, Torrance, CA 90502, USA; 2 Harbor-UCLA Medical Center, Division of Infectious Diseases, Torrance, CA 90502, USA; 3 Department of Medicine, David Geffen School of Medicine at UCLA, Los Angeles, CA 90024, USA; 4 The Lundquist Institute for Biomedical Innovation at Harbor-UCLA Medical Center, Institute for Infection and Immunity, Torrance, CA 90502, USA; 5 Division of Infectious Diseases and International Health, Department of Medicine, Duke University School of Medicine, Durham, NC 27708, USA; 6 Duke Clinical Research Institute, Durham, NC 27708, USA; 7 School of Medicine and Public Health, University of Wisconsin, Madison, WI 53726, USA

**Keywords:** *Staphylococcus aureus*, virulence, resistance, small colony variant, host defense peptide

## Abstract

Background: *Staphylococcus aureus* bacteremia (SAB) is a common and life-threatening bloodstream infection often caused by methicillin-resistant SA (MRSA) isolates. Up to 35% of SAB patients fail to clear infection with gold-standard anti-MRSA antibiotics, even if the isolate meets susceptibility breakpoints in conventional assays in vitro. Such outcomes are termed persistent and may involve small colony variant (SCV) adaptation of SA in vivo. Methods: In this study, we assessed virulence phenotypes and mechanisms in persistent (PB) vs. resolving (RB) MRSA isolates from SAB. Results: Overall, PB isolates caused less hemolysis or biofilm formation than RB isolates, but proteolysis was equivalent. Attenuation of these virulence phenotypes increased longitudinally during the course of SAB. Although PB vs. RB isolates had similar human endothelial cell invasion rates, PB isolates more frequently formed SCVs intracellularly and inversely correlated with pH. Study PB and RB isolates exhibited distinct susceptibilities to prototypic human host defense peptides (HDPs), which were influenced by antibiotics and pH. Furthermore, mechanistic signatures of HDPs differed between PB and RB isolates. Conclusions: Together, these results reveal that MRSA isolates from PB vs. RB outcomes of SAB have differential virulence profiles that suggest coordinated immune subversion in PB. Understanding MRSA adaptations that promote persistence in SAB may enable innovative agents and strategies to address these challenging infections.

## 1. Introduction

*Staphylococcus aureus* (SA) is the most common etiologic agent of bacteremia and metastatic sequelae [1,2,3]. In methicillin-resistant SA (MRSA) bacteremia, up to 35% of patients succumb even on gold-standard antibiotic therapy, equating to 20,000 deaths/year in the U.S. alone [4,5]. While persistent MRSA isolates are susceptible to antibiotics in vitro, antibiotic therapy fails to clear these isolates from the bloodstream despite appropriate pharmacological profiles [6,7]. This survival of MRSA in vivo despite antibiotic susceptibility in vitro is termed persistence. Persistence after just 1 day of appropriate therapy correlates with worsened outcomes and increased antibiotic usage [8,9]. In turn, escalation of antibiotic usage promotes adverse events and increased antibiotic resistance [2,10,11,12]. This vicious cycle of persistence, antibiotic escalation and antibiotic resistance is a growing public health concern [13]. Further, metastatic complications, length of hospital stay and mortality progressively increase as a function of bloodstream persistence even on appropriate therapy [14,15]. Unfortunately, despite meritorious efforts, there remains no FDA-approved vaccine for the prevention or mitigation of SA infection [15,16]. Collectively, these issues urge a real-world understanding of virulence and resistance characteristics associated with MRSA persistence. This knowledge should afford new strategies to prevent and treat life-threatening persistent infections.

Several lines of evidence suggest that SA adapts its virulence phenotypes during bloodstream infection to promote persistent bacteremia [17,18,19,20,21]. We hypothesize that persistent outcomes occur when MRSA encounters host immune effectors in the context of antibiotics. Here, MRSA may undergo genotypic and phenotypic changes that result in the small-colony variant (SCV) phenotype with enhanced ability to evade or subvert immune barriers (e.g., invade host cells) and/or reduced susceptibility to host defense peptides (HDPs) or antibiotics [22,23,24,25]. The current study explores virulence and immune subversive phenotypes among MRSA clinical isolates from human persistent bacteremia (PB; culture positive after 3 days of vancomycin therapy) vs. resolving bacteremia (RB; culture negative within 3 days of vancomycin therapy). The key virulence phenotypes (virulotypes) studied in this respect were hemolysis, proteolysis, biofilm formation, host-cell invasion and intracellular survival, SCV adaptation and susceptibility, and mechanistic profiles of prototypic host defense peptides.

## 2. Results

### 2.1. Antibiotic Susceptibility

**Figure 1:**
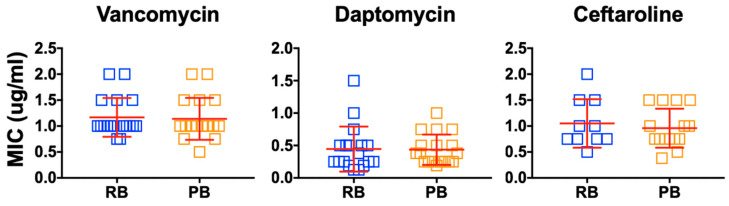
Distribution of antibiotic susceptibility among MRSA isolates from human bacteremia. Vancomycin, daptomycin or ceftaroline were evaluated using the E-test. The rationale and number of isolates studied are detailed in Methods. PB, persistent bacteremia MRSA isolate; RB, resolving bacteremia MRSA isolate.

Prior studies from our group identified a set of 75 persistent (PB) vs. resolving (RB) bacteremia MRSA isolates from closely matched patients [26,27,28]. Minimum inhibitory concentrations (MICs) of vancomycin, daptomycin and ceftaroline were determined. As summarized in Figure 1, no significant differences in antibiotic susceptibility were detected in PB vs. RB isolates for any antibiotic tested in vitro.

### 2.2. Virulence Phenotypes

**Figure 2:**
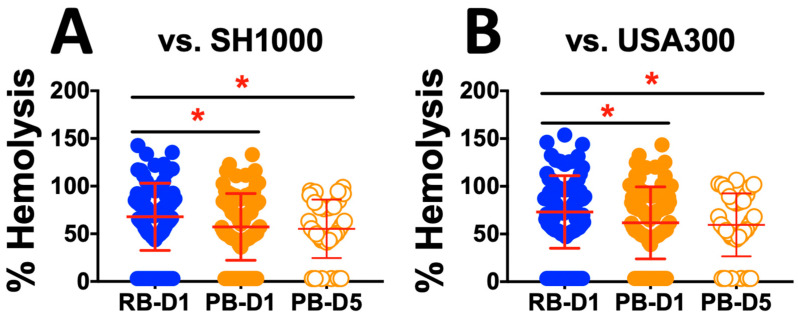
Distribution of hemolysis among MRSA isolates from human bacteremia. Hemolysis caused by RB or PB isolates was normalized to reference strains SH1000 (**A**) or LAC (**B**). Initial presenting isolates (RB-D1 and PB-D1) and day 5 PB isolates (PB-D5) were evaluated. Values are expressed as a percentage of the respective control (SH1000 or LAC) and represented as the mean of triplicate experiments (*n* = 3). * *p* < 0.05.

**Hemolysis.** Isolates from day 1 (D1) and day 5 (D5) of PB and isolates from D1 of RB were assessed for hemolysis on sheep and rabbit blood agar. Analyzed as cohorts, PB-D1 and PB-D5 isolates were significantly less hemolytic than RB-D1 isolates when normalized to either reference strains SH1000 or LAC (*p* < 0.05; Figure 2). Overall, PB-D1 and PB-D5 isolates were 9% and 17% less hemolytic than RB-D1 isolates, respectively.

**Figure 3:**
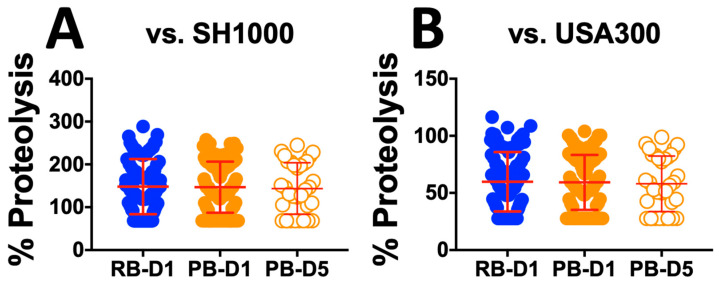
Distribution of proteolysis among MRSA isolates from human bacteremia. Proteolysis caused by RB or PB isolates was normalized to reference strains SH1000 (**A**) or LAC (**B**). Initial presenting isolates (RB-D1 and PB-D1) and day 5 PB isolates (PB-D5) were evaluated. Values are expressed as a percentage of the control (SH1000 or LAC) and represented as the mean of triplicate experiments (*n* = 3).

**Proteolysis.** In parallel, RB and PB isolates were evaluated for proteolysis on casein agar per our previously reported methods [29]. Analyzed as cohorts, there were no differences between RB vs. PB isolates when normalized to SH1000 or LAC (Figure 3).

**Figure 4:**
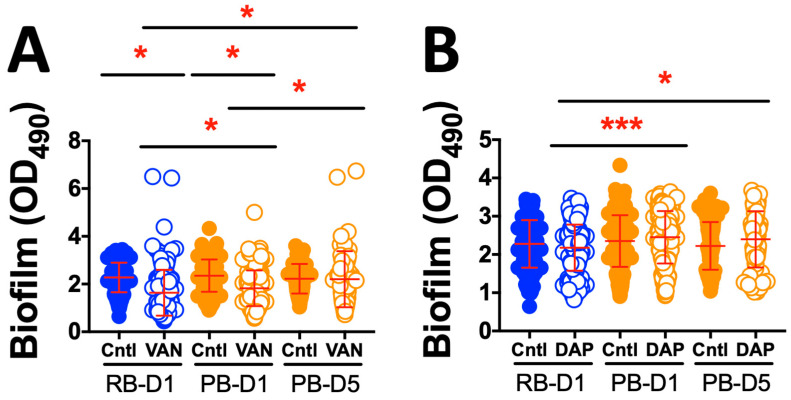
Impact of antibiotics on biofilm formation among MRSA isolates from human bacteremia. (**A**) Vancomycin (VAN); (**B**) Daptomycin (DAP). Biofilm matrices generated by RB or PB isolates were measured by relative optical density at 490nm (OD_490_) [29]. Initial presenting isolates (RB-D1 and PB-D1) and day 5 isolates from PB (PB-D5). Each data point represents the mean of absolute values from triplicate experiments ± standard deviation (*n* = 3). Cntl: no antibiotic control. * *p* < 0.05; *** *p* < 0.001.

**Biofilm.** Additionally, RB and PB isolates were assessed for biofilm matrix production in the presence and absence of vancomycin (VAN) or daptomycin (DAP) (Figure 4). Biofilm formation under VAN exposure was significantly greater in PB-D1 than in RB-D1. Further, biofilm formation under VAN exposure was significantly greater in PB-D5 than in RB-D1 or PB-D1. Significant reductions in biofilm formation comparing control vs. VAN exposure were observed at D1 for RB and PB isolates but not at D5 for PB isolates. By comparison, biofilm formation under DAP exposure was significantly greater in PB-D1 than in RB-D1. Further, biofilm formation under DAP exposure was significantly greater in PB-D5 than in RB-D1. DAP exposure was not associated with reduced biofilm formation in PB-D5 as compared to PB-D1. No significant reductions in biofilm formation comparing control vs. DAP exposure were observed. Collectively, this pattern of data indicates that PB isolates adapt more robustly than RB isolates to VAN or DAP exposure as manifested by increases in biofilm formation over time.

### 2.3. Small Colony Variants

**Figure 5:**
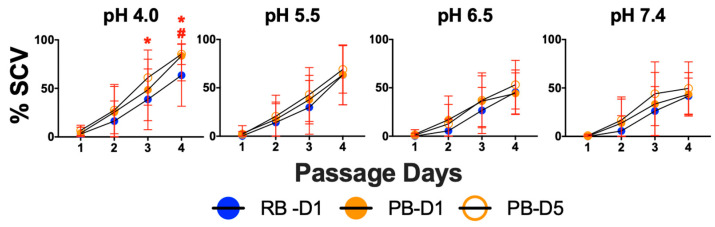
Impact of pH and time on SCV formation in MRSA isolates. PB and RB isolates were passaged in vitro as described in Section 4. Data represent the mean (±standard deviation) of 10 or more RB-D1, PB-D1 and PB-D5 isolates tested independently (*n* ≥ 2 replicates). Values are expressed as a percentage of the control at study onset (day 0). * *p* < 0.05 PB-D5 vs. RB-D1; **#** *p* < 0.05 PB-D1 vs. RB-D1.

Adaptation to the small colony variant (SCV) phenotype is known to be associated with reduced antibiotic susceptibility and immune clearance in MRSA infection. We evaluated the propensity of PB vs. RB isolates from human bacteremia to form SCVs in conditions reflecting the range of pH relevant to extracellular vs. intracellular microenvironments of human phagocytes (Figure 5).

**SCV Frequency.** The PB and RB isolate set was screened for formation of SCVs in minimal essential media (MEM) at pH 4.0, 5.5, 6.5 and 7.4 over 4 days. Overall, MRSA isolates formed more SCVs as an inverse function of pH regardless of their PB or RB classification. Furthermore, more SCVs were formed over time for either category. A significant difference was detected in SCV formation at pH 4, day 3, between PB-D5 and RB-D1 isolates. This significant difference expanded to PB-D5 or PB-D1 vs. RB-D1 isolates at day 4. No significant difference was observed between PB-D5 and PB-D1 under any condition. This pattern of findings suggests PB isolates are more proficient at adapting to SCV phenotypes at pH values relevant to intracellular microenvironments and over longer time periods.

**Figure 6:**
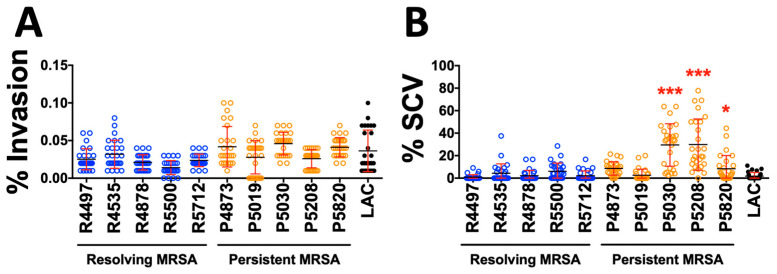
Differential host cell interactions of PB vs. RB isolates. (**A**) Percentage of SA inoculum that invaded hTERT cells (host cell invasion). (**B**) Percentage of intracellular SA that undergoes SCV adaptation (SCV propensity). Values are expressed as a percentage of the control and represented as the mean of triplicate experiments (*n* = 3). * *p* < 0.05; *** *p* < 0.001 as compared to all RB isolates, one-way ANOVA.

**Host Cell Interaction.** Based on the hemolysis, proteolysis, biofilm and SCV results above, we curated five highly-matched PB and RB isolate pairs for further characterization of host cell interaction (Table 1; Methods). Human endothelial cells (hTERT) were subjected to infection with PB or RB isolates (see Section 4), and percent invasion and intracellular SCV formation were quantified at 1 or 3 h post-inoculation, respectively. As comparative cohorts, no significant differences were observed in hTERT cell invasion among PB and RB isolates or reference strain LAC USA300 (Figure 6A). However, among the intracellular survivors, a majority of PB isolates formed significantly more SCVs during intracellular invasion than RB isolates or reference strain LAC USA300 (Figure 6B).

### 2.4. Host Defense Peptides Interactions

**Figure 7:**
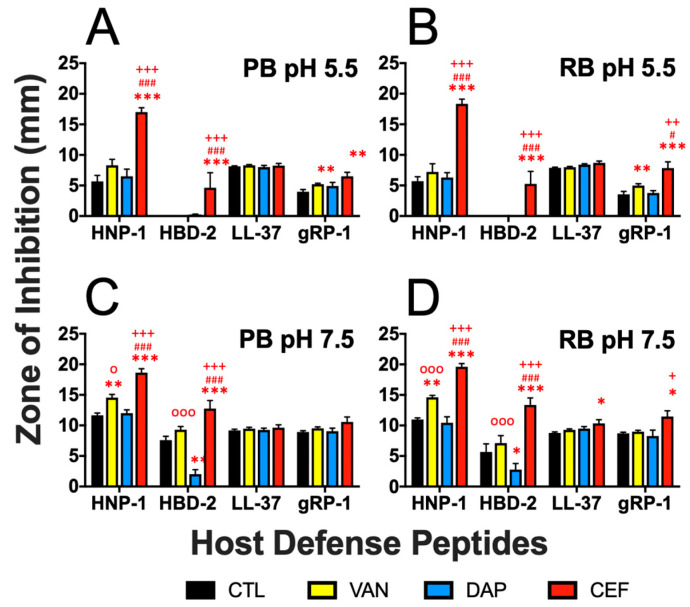
Susceptibility of PB vs. RB isolates to HDPs in the presence or absence of antibiotics. HDP susceptibility with or without antibiotics was quantified using our established radial diffusion assay (see Section 4). (**A**) PB isolates at pH 5.5; (**B**) RB isolates at pH 5.5; (**C**) PB isolates at pH 7.5; (**D**) RB isolates at pH 7.5. Histograms represent pooled data of 5 PB vs. 5 RB isolates (*n* = 3 replicates each). Control (Ctl), vehicle only; VAN, vancomycin; DAP, daptomycin; CEF: ceftaroline, HNP-1: human neutrophil peptide-1 (granulocyte); HBD-2, human beta defensin-2 (epithelium); LL-37, cathelicidin (granulocyte and epithelium); gRP-1, gamma RP-1 (platelet). * *p* < 0.05, ** *p* < 0.01; *** *p* < 0.001 antibiotic vs. control; ° *p* < 0.05, °°° *p* < 0.001 VAN vs. DAP; ^#^ *p* < 0.05, ^###^ *p* < 0.001 VAN vs. CEF; ^+^ *p* < 0.05, ^++^ *p* < 0.01, ^+++^ *p* < 0.001 DAP vs. CEF. Statistical analyses were performed by t-test with Holm-Sidak correction for multiple comparisons.

The ability to survive in the face of host defense peptides (HDPs) is a hallmark MRSA virulence phenotype reflecting immune evasion. We evaluated the propensity of PB vs. RB isolates for their susceptibility to prototypic human HDPs representing granulocyte-, epithelial- and platelet-derived host defense. Assays were performed in the presence and absence of gold-standard anti-staphylococcal antibiotics (VAN, DAP or ceftaroline [CEF]) at pH conditions representing extracellular and intracellular immune microenvironments (Figure 7). In parallel, mechanistic fingerprinting of these HDPs compared to antibiotics was performed on prototypic PB vs. RB isolates (Figure 9).

**Figure 8:**
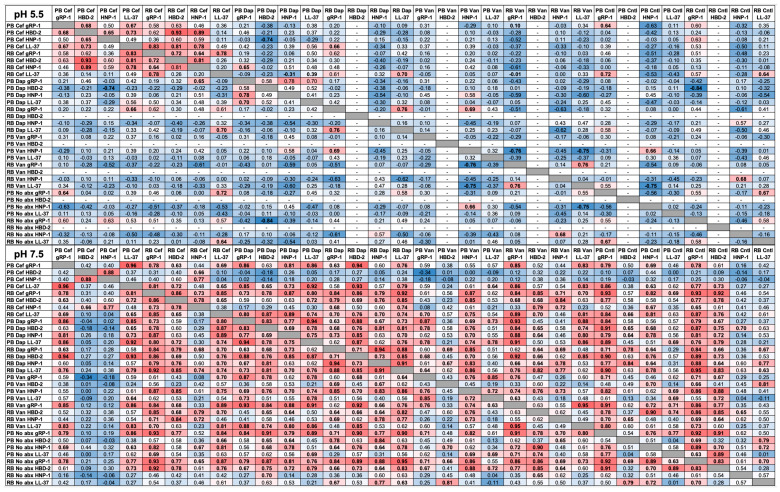
Pearson correlation of PB vs. RB susceptibility to HDPs in the presence or absence of antibiotics. (**A**) pH 5.5; (**B**) pH 7.5. Relationships were determined using Pearson linear correlation analysis (*n* = 10). Values represent correlation coefficients (R; distribution −1 to 1). Blue values indicate inverse correlations (R < 0.5), whereas red values indicate direct correlations (R > 0.5). Bolded values represent significance (*p* < 0.05; Supplemental Table S1). Abbreviations are defined in text. Dashes in columns and rows indicate no correlation determinable due to lack of efficacy.

**HDP Susceptibility**. Study HDPs exerted significant antimicrobial activity against MRSA isolates in vitro. As anticipated, HDPs generally had greater efficacy at pH 7.5 against PB or RB isolates than at pH 5.5. Furthermore, specific HDPs in combination with specific antibiotics exhibited differential patterns of efficacy against PB and RB isolates at distinct pH (Figure 7 and Figure 8; Supplemental Table S1). Themes and patterns of susceptibility to HDPs with or without antibiotics, and at pH 5.5 vs. pH 7.5, are summarized below and visualized in Figure 8.

**HNP-1**: Human neutrophil peptide-1 (HNP-1) is a defensin localized to neutrophils and functions in the acidic phagolysosome [30]. CEF achieved significant HNP-1 potentiation against PB and RB isolates at both pH 5.5 and 7.5 compared to HNP-1 alone or in combination with VAN or DAP. VAN potentiated HNP-1 in PB and RB isolates only at pH 7.5. DAP did not significantly potentiate HNP-1 against any isolate at either pH. No antibiotic antagonized HNP-1 in any condition.

**HBD-2**: Human beta defensin-2 (HBD-2) is a defensin localized to epithelium and functions at the barrier interface where pH can vary [31,32,33]. CEF achieved significant HBD-2 potentiation against PB and RB isolates at both pH 5.5 and 7.5 compared to HBD-2 alone or in combination with VAN or DAP. Conversely, DAP antagonized HBD-2 at pH 7.5 against PB and RB isolates. VAN did not significantly affect HBD-2 efficacy against any isolate at either pH.

**LL-37**: LL-37 is a 37-amino acid, leucine dyad-containing cathelicidin localized to neutrophils and epithelium and functions across a range of immune contexts and pH [34,35]. No antibiotic potentiated the efficacy of LL-37, with the exception of CEF against RB isolates at pH 7.5. No antibiotic antagonized LL-37 in any condition.

**gRP-1**: Gamma RP-1 (gRP-1) is an engineered synthetic congener of platelet CXCL4 and functions in blood matrices (whole blood, plasma and serum) and was evaluated for antimicrobial activity at pH 7.5 and pH 5.5 [36]. VAN and CEF achieved significant gRP-1 potentiation against PB and RB isolates at both pH 5.5 compared to gRP-1 alone. CEF achieved significant gRP-1 potentiation against RB but not PB isolates at pH 7.5. VAN did not exhibit this effect. Furthermore, DAP did not potentiate the efficacy of gRP-1, and no antibiotic antagonized gRP-1 under any condition.

**HDP Mechanisms**. Multiparameter mechanistic fingerprinting was performed to evaluate six distinct mechanisms of anti-staphylococcal activity simultaneously against a target isolate as previously described [37]. Mechanisms of action evaluated included: (1) osmodisruption (forward scatter [FSC]; cellular swelling); (2) intracellular nucleic acid condensation/cytoplasmic refractivity (side scatter [SSC]; granularity); (3) perturbation of cell membrane (CM) energetics (ENR) (e.g., transmembrane potential); (4) CM permeabilization (PRM); (5) annexin V binding (ANX) (negatively charged phospholipids and CM turnover); and (6) caspase-like/metacaspase-like cell death protease induction (CSP) [37]. Resulting mechanigrams visualize each mechanism relative to the others in the context of isolate exposure to HDPs or control antibiotics.

**Figure 9:**
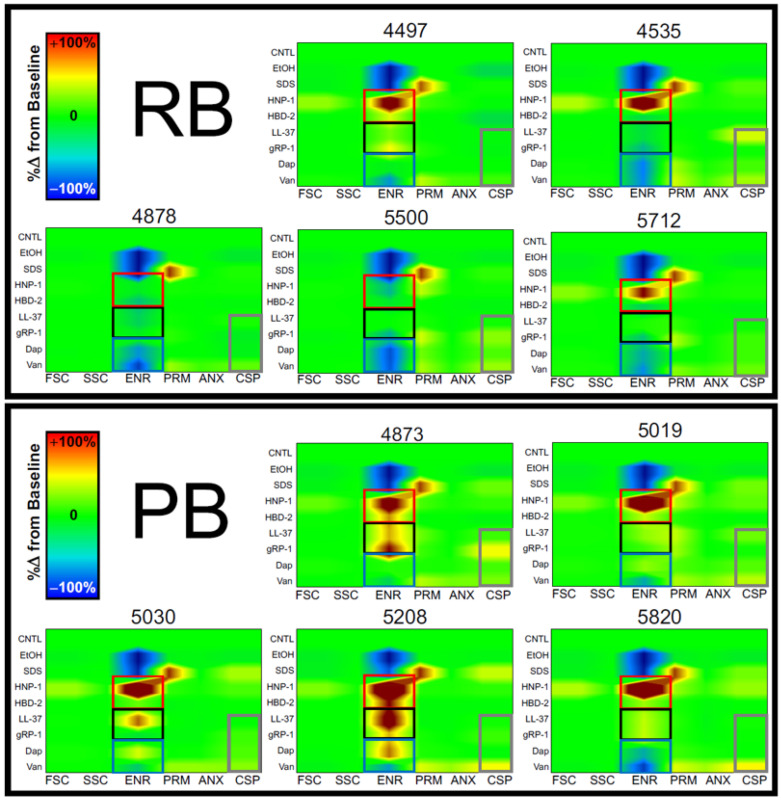
Distinct mechanistic fingerprints in PB and RB isolates due to HDPs and antibiotics. Exposures were conducted at pH 7.5, given the outcomes of the susceptibility testing (see Figure 7). Results are as described in the text. Values are expressed as a percentage of the control and represented as the mean of multiple experiments. CNTL: untreated control; EtOH: ethanol; SDS: sodium dodecyl sulfate; HNP-1, human neutrophil peptide-1; HBD-2, human beta defensin-2; LL-37, cathelicidin; gRP-1, gamma RP-1; Dap, daptomycin; Van, vancomycin; FSC, forward scatter; SSC, side scatter; ENR, energetics; PRM, permeabilization; ANX, annexin V binding; CSP, caspase-like protease induction. Categories: defensins (red boxes); cathelicidin and CXCL4 congener (black boxes); antibiotics (blue boxes); selected HDPs and antibiotics (gray boxes). *p*-values are summarized in Supplemental Table S2.

MRSA isolates exhibited distinct mechanistic signatures when exposed to HDPs or antibiotics (Figure 9). However, several themes emerged from these results, including significant differences between PB vs. RB isolates (see Supplemental Table S2). Defensin HNP-1 hyperpolarized (increased ENR) all PB isolates, whereas only a subset of RB isolates exhibited this effect (red boxes). In contrast, defensin HBD-2 significantly increased ENR in a subset of PB isolates but not in RB isolates (red boxes). Interestingly, LL-37 exerted significant increases in ENR in PB isolates, but it had no substantive impact on ENR in any RB isolates (black boxes). Only one RB isolate was modestly hyper-energized by gRP-1, whereas most PB isolates had increased ENR (black boxes). All isolates had lower ENR due to VAN; this theme was more prominent in RB than PB isolates. DAP de-energized all but one RB isolate, but only one PB isolate (blue boxes). In contrast, DAP hyper-energized (increased ENR) a subset of PB isolates, but no RB isolate. These differences in Dap-altered energetics were significantly different between PB and RB. VAN induced PRM to an equivalent degree in PB and RB isolates. gRP-1 induced modest PRM in RB isolates, but it had no substantive effect on PRM in PB isolates. While no condition resulted in increased ANX signal in RB isolates, increases were observed in all PB isolates; this difference was statistically significant. Consistent with ANX findings, all but one PB isolate exhibited significant induction of CSP in response to one or more HDP or Van, while only one RB isolate exhibited this effect (gray boxes). By comparison, DAP did not induce CSP in any isolate tested. gRP-1 and LL-37 induced modest changes in CSP that were variable in RB or PB isolates (gray boxes). While HNP-1 induced modest increases in FSC, these were equivalent in RB and PB isolates. No HDP significantly increased in FSC or SSC in any isolate studied. Affirming the veracity of these results, control conditions yielded expected outcomes: (1) untreated control exhibited no change in any mechanism of action; (2) ethanol significantly lowered ENR in all isolates studied; (3) SDS lowered ENR in all isolates; and (4) the detergent sodium dodecyl sulfate (SDS) caused extensive PRM in all isolates. Statistical analyses are provided in Supplemental Table S2.

## 3. Discussion

Outcomes of MRSA bacteremia undoubtedly involve complex interactions among the host, pathogen and antibiotic therapy [38,39,40]. In persistent MRSA bacteremia (PB), the host bloodstream remains culture positive even when gold-standard anti-MRSA antibiotics are appropriately used. This PB outcome implies inadequate host immunity, immune subversion and/or antibiotic resistance of the organism. In the current study, we evaluated PB and resolving MRSA bacteremia (RB) isolates to compare their virulence phenotypes. Several interesting findings emanated from this work.

To obviate the potential that in vitro antibiotic susceptibility alone explains outcomes, we performed conventional testing for susceptibility to vancomycin (VAN), daptomycin (DAP) or ceftaroline (CEF) on all study isolates. Overall, no significant differences were detected in minimum inhibitory concentration (MIC) between PB vs. RB isolates, with all isolates falling within susceptibility breakpoints (Figure 1). These results are consistent with prior reports in which MRSA bacteremia isolates exhibit equivalent antibiotic susceptibility regardless of clinical outcomes [41,42,43].

We next examined the propensity for isolates to cause hemolysis relative to two well-characterized laboratory reference strains (LAC-USA300 and SH1000). In addition, SAB isolates from day 1 and day 5 of PB were compared to day 1 of RB. Overall, the day 1 RB isolates exhibited greater hemolytic capability than day 1 or day 5 PB isolates. However, isolates from day 1 and day 5 PB patients had equivalent hemolytic capabilities (Figure 2). These findings support our hypothesis that PB isolates subvert host defense through an intentional lack of lytic effects on host cells, enabling their stealth [44,45]. Similarly, we compared the PB and RB isolate cohorts for their propensity to exert proteolysis in vitro. In contrast to hemolysis, no significant differences in proteolytic activity were found among any comparison of the PB vs. RB isolates (Figure 3). In parallel, biofilm production was compared for PB and RB isolates with or without VAN or DAP in vitro. Overall, there was no difference detected in biofilm formation in the absence of antibiotics. Interestingly, VAN yielded lower biofilm formation in day 1 RB and day 1 PB isolates but not day 5 PB isolates (Figure 4A). In contrast, day 1 RB isolates produced significantly less biofilm in the presence of DAP than either day 1 or day 5 PB isolates (Figure 4B). This pattern of results is consistent with phenotypic plasticity, particularly in PB isolates over the course of infection in the presence of antibiotics [46,47]. Collectively, these results suggest that PB isolates are adapted for reduced virulence phenotypes as a persistence strategy, particularly in the presence of antibiotics [48,49,50]. These findings are consistent with prior reports, which documented virulence adaptations of MRSA during infection in the setting of VAN therapy [46].

Next, we assessed the propensity for PB vs. RB isolates to adapt to small colony variant (SCV) phenotypes as a function of time and pH (Figure 5) [51]. Compared to RB day 1, PB day 1 or day 5 isolates yielded a significantly greater percentage of SCVs at days 3 and 4 of serial passage in vitro. This effect was only detectable at pH 4.0. Given that serum and the intracellular milieu of granulocyte phagolysosomes are both acidic, these findings support the hypothesis that PB isolates exploit acidic microenvironments for SCV adaptation [52,53,54]. Furthermore, these observations substantiate the concept that SCV adaptation is a temporal process, increasing as a function of time. Our current and previously reported effects of pH on SA virulence and immune subversion [55,56,57] have been recently supported by other work [58,59,60]. For example, prior reports emphasized that the microenvironment within target host cells induces small colony variant formation in SA [19,61,62]. Mechanisms posited as responsible for such induction include the redox state, phagolysosomal milieu and limiting nutritional availability within the infected host cell. These hypotheses harmonize with data from previous studies corresponding to these mechanisms [18,19].

In related experiments, five prototypic PB vs. RB isolates from well-matched cases were assessed for their ability to invade and undergo SCV adaptation within human endothelial cells, as evaluated in vitro (Figure 6). We detected no significant difference in isolate capacity for host cell invasion. However, once internalized, the majority of PB isolates underwent significantly greater frequency of SCV adaptation than RB isolates. These findings are consistent with our hypothesis that PB isolates subvert the intracellular microenvironments of host cells as a persistence strategy. These observations are consistent with intracellular SCV adaptation of *S. aureus* in macrophages [63,64,65,66].

Another interesting aspect of the current study was a comparison of PB vs. RB isolate susceptibility to representative host defense peptides (HDPs) in the presence or absence of anti-staphylococcal antibiotics (Figure 7). These HDPs were selected for their comparative sources of human neutrophils, human epithelium or platelets [67]. Furthermore, these assays were performed at pH conditions to mimic acidic intracellular (pH 5.5) vs. neutral bloodstream contexts (pH 7.5) [56]. Overall, isolates exhibited greater susceptibility to all HDPs at pH 7.5 vs. pH 5.5. This difference was particularly apparent when comparing the defensins HNP-1 and HBD-2 at pH 5.5. Another overarching theme was the significant potentiation of all HDPs by CEF against at least some study isolates. These in vitro data are in alignment with the efficacy of CEF in salvage therapy for persistent MRSA bacteremia that has failed VAN and DAP [68,69,70,71]. In contrast, DAP did not potentiate any HDPs tested against PB or RB isolates at either pH, and antagonized HBD-2 at pH 7.5 in PB and RB isolates. This latter observation is unsurprising, given that DAP functions mechanistically in ways similar to HDPs [72,73,74]. VAN significantly potentiated the defensins at pH 7.5 but not at pH 5.5. Once again, these results are congruent with the basic premise that PB isolates subvert host immunity and antibiotic therapy using strategies that exploit acidic microenvironments in the host. These findings are supported by recent studies demonstrating that SA evades immunity by generating or exploiting acidic host contexts [69,75,76].

To further explore specific HDP interactions with PB vs. RB isolates, we performed mechanistic fingerprinting using six-parameter multi-color flow cytometry (Figure 9) [37,77]. Overall, PB isolates exhibited significant cell membrane hyper-energization in response to HNP-1 and, to a lesser extent, other HDPs. In contrast, RB isolates exhibited significant hypo-energization of the cell membrane upon exposure to Van and, to a lesser extent, DAP. This pattern of results suggests cell energetics are a distinguishing feature of PB vs. RB isolates in the face of anti-infective pressures. It should be emphasized that the majority of PB isolates exhibited characteristics of programmed cell death mechanisms on exposure to HDPs [37]. This theme was generally not observed for RB isolates. Collectively, this finding supports our hypothesis that PB isolates strategically exploit intracellular environments to escape HDPs that would induce regulated cell death. Finally, it is important to note that no two isolates had identical mechanistic fingerprints. This finding underscores the probability that each case of human MRSA bacteremia is a unique intersection among the specific isolate, host and antibiotic [39,40,78].

We emphasize the clinical relevance of the study isolates regarding the observed experimental outcomes. All isolates were obtained from MRSA bacteremia patients appropriately treated with Van. Persistence was defined by culture positivity within five days of VAN therapy [79], whereas resolution corresponded to culture-negative outcomes within one day of therapy. In this context, the current findings offer plausible host—MRSA relationships that may shape PB vs. RB outcomes: (1) PB isolates appear to be less virulent as a persistence strategy; (2) PB isolates appear to exploit acidic microenvironments for enhanced SCV adaptation; (3) PB isolates may have greater propensity for SCV formation within non-professional phagocytes such as endothelial cells; and/or (4) VAN may impose selection pressures in vivo that favor these individual or collective immune subversion strategies resulting in persistence.

It should be understood that the current studies have important limitations. Whether these in vitro findings hold true in vivo has not been fully established. However, our studies attempted to create conditions that simulate the human host in terms of pH, relevant HDPs and use of FDA-approved anti-MRSA antibiotics. We recognize that adaptation to an SCV phenotype in vivo is a complex and multi-factorial process that may not be fully recapitulated in vitro. For example, we and others have observed that PB isolates undergo substantial genomic rearrangements in vivo that may revert once the organism undergoes culturing in artificial media in vitro [17]. Nonetheless, by determining biomarkers that may distinguish PB from RB isolates in vitro, it may be possible to create novel assays to prospectively predict and intervene in patients likely to have persistent outcomes if treated conventionally. In summary, while the methods used may not fully mirror human disease, they almost certainly shed new light on host–pathogen relationships integral to shaping the balance of infection and immunity.

## 4. Materials and Methods

### 4.1. Staphylococcus aureus Strains

This study utilized 75 different *S. aureus* clinical isolates obtained from the *Staphylococcus aureus* Bacteremia Group (SABG) repository at Duke University Medical Center (Durham, NC, USA) (Table 1) [26,78]. In hemolysis, proteolysis and biofilm assays, the entire isolate set was studied. For MIC, host-cell invasion, SCV formation, and host defense peptide susceptibility and mechanistic fingerprinting studies, a subset of this isolate library was used as detailed in the Results. For consistency in the latter studies, the same well-characterized organism set was used based on their representing prototypic PB vs. RB isolates. Control SA strains included LAC, a USA300 MRSA isolate [80], and SH1000, a well-characterized MSSA isolate [81]. Organisms from master cell banks were cultured to log-phase in brain heart infusion (BHI; Becton Dickinson (BD), Difco, Franklin Lakes, NJ, USA) medium at 37 °C. Resulting cells were harvested, washed in phosphate-buffered saline (PBS; pH 7.2; BD Difco, Franklin Lakes, NJ, USA), sonicated, quantified by spectrophotometry, and diluted to the desired inoculum in PBS buffer. Isolates prepared in this manner were used as inocula for specific assays, detailed below.

**Table 1:** *Staphylococcus aureus* strains used in this study.

Strain	Description	Source
R4497	Resolving bacteremia clinical isolate	[26]
R4535	Resolving bacteremia clinical isolate	[26]
R4878	Resolving bacteremia clinical isolate	[26]
R5500	Resolving bacteremia clinical isolate	[26]
R5712	Resolving bacteremia clinical isolate	[26]
P4873	Persistent bacteremia clinical isolate	[26]
P5019	Persistent bacteremia clinical isolate	[26]
P5030	Persistent bacteremia clinical isolate	[26]
P5208	Persistent bacteremia clinical isolate	[26]
P5820	Persistent bacteremia clinical isolate	[26]
LAC	CA-MRSA USA300 isolate; Los Angeles County	[82]
SH1000	Laboratory strain 8325-4 with repaired *rsbU* mutation	American Type Culture Collection

### 4.2. Antibiotics and Host Defense Peptides

Vancomycin (VAN) (Sigma Aldrich, St. Louis, MO, USA), daptomycin (DAP) (Merck, Rahway, NJ, USA) and ceftaroline (CEF) (Forest Laboratories, New York City, NY, USA) were dissolved in double-distilled water (ddH20). Host defense peptides (HDPs) representing relevant immunologic contexts were studied. Unless otherwise stated, peptides used in this study were obtained from Peptides International, Louisville, KY, USA. Human neutrophil peptide 1 (hNP-1) is a prototypic α-defensin found in human neutrophil granules [30]. Human β-defensin 2 (hβD-2) is a predominant peptide elaborated by epithelial tissues throughout the body [31,32,33]. LL-37 is a human cathelicidin expressed in epithelial tissues and neutrophils [34,35]. The mimetic peptide gamma-RP-1 (gRP-1) is a synthetic congener engineered in part from CXCL4 kinocidins. This peptide was synthesized, purified, and authenticated as previously detailed [36]. Each study peptide has demonstrated in vitro antimicrobial activity against SA [37,56,57,77,83,84,85].

### 4.3. Minimum Inhibitory Concentration

Minimum inhibitor concentrations (MICs) of antibiotics against SA isolates were determined using the recommended standard Clinical Standards Laboratory Institute (CLSI) gradient concentration strip (E-test; BioMerieux, Craponne, France) protocol (range 0.016–256 μg/mL) [27,86]. In VAN and DAP MIC assays, 18 PB and RB isolates were analyzed. In CEF MIC assays, 10 RB and 15 PB isolates were analyzed. Isolates were cultured in Mueller–Hinton broth and plated onto cation-adjusted Mueller–Hinton agar, followed by placement of an E-test strip. Cultures were incubated overnight at 37 °C, and the lowest concentration inhibiting growth was recorded as the MIC. Assays were repeated a minimum of 3 times (*n* = 3+) on different days for experimental validation.

### 4.4. Hemolysis Assay

Strains were grown in Brain–Heart Infusion broth (BHI; BD Difco, Franklin Lakes, NJ, USA) and inoculated (10^6^ CFU/10μL) by microdrop plating onto tryptic soy agar containing 5% rabbit blood (Remel, Lenexa, KS, USA). Plates were incubated at 37 °C for 24 h, followed by cold shock at 4 °C for 48 h. Cold shocking reveals hemolytic activity for ease of visualization and measurement [87,88,89]. Diameters (mm^2^) of zones of clearance were measured, and data represented as a percentage of control (LAC-USA300 or SH1000). The results were normalized by comparing the zone of hemolysis for an experimental isolate to that caused by these two well-characterized reference strains (experimental zone/reference zone; expressed as percent). As a standard practice, hemolysis was determined on 5% sheep blood agar. Because certain SA hemolysins preferentially lyse other mammalian red blood cells, the results were verified using 5% rabbit blood agar and confirmed comparable results [90]. Significant differences among outcomes were analyzed using Student’s t-test.

### 4.5. Proteolysis Assay

Strains were grown in BHI and plated (10^6^ CFU/10μL), as above, onto casein agar (1% *w*/*v*) [91]. Plates were grown at 37 °C for 24 h. Zones of proteolysis (mm^2^) were measured and normalized to reference controls. Here, the results were normalized by comparing the zone of proteolysis for an experimental isolate to that caused by these two well-characterized reference strains (experimental zone/reference zone; expressed as percent). Data are represented as a percentage of the control (LAC-USA300 or SH1000). Significant differences among outcomes were analyzed using Student’s t-test as detailed above.

### 4.6. Biofilm Assay

Strains were grown in BHI with 0.5% glucose with or without antibiotics for 18 h at 37 °C in 96-well flat-bottom polystyrene plates (Fisher Scientific, Waltham, MA, USA) [92,93]. VAN and DAP were selected for these studies because they represent the first-line therapies often used in treating clinical MRSA bacteremia. After removing culture suspensions, plates were washed with 1X PBS and dried at 37 °C for 1 h. Biofilms were stained with 0.1% safranin (Fisher Scientific, Waltham, MA, USA), washed with distilled water, and decolorized with 30% glacial acetic acid (Fisher Scientific, Waltham, MA, USA). The resulting biofilm density was then measured by spectrophotometry at OD_490_ and compared to the no-antibiotic control.

### 4.7. Emergence of SCVs

SA isolates were systematically passaged for 4 days (37 °C) across a range of pH from 4.0 to 7.4 in minimal essential medium (MEM) titrated with hydrochloric acid. At each time point, samples were quantitatively cultured by plating onto 2% sheep blood agar and enumerated for survival and percent SCV formation.

### 4.8. Endothelial Cell Invasion Assay

MRSA isolates were assayed for invasion and SCV formation within human telomerase reverse transcriptase (hTERT)-immortalized dermal microvascular endothelial cells (ATCC; Manassas, VA, USA). MRSA was grown in BHI to log phase, washed, and resuspended in RPMI (Fisher Scientific, Waltham, MA, USA) to 1 × 10^8^ CFU/mL. hTERT cells were washed with RPMI alone, and bacteria were added to each well for a multiplicity of infection (MOI) of 1:20. Cells were incubated for 30 min at 37 °C with 5% CO_2_. Lysostaphin (Fisher Scientific, Waltham, MA, USA) was added to kill extracellular bacteria (4 U/mL, 30 min post-infection). Cells were harvested at 1 and 3 h post-infection to determine invasion and intracellular survival, respectively. To do so, host cells were lysed with 9 mL of ddH_2_O and bacteria rescued with 1 mL of 10× PBS (Fisher Scientific, Waltham, MA, USA). Supernatants were plated onto blood agar plates to enumerate CFU. At 1 h post-infection, the percent of inoculum internalized was quantified. On average, each hTERT cell contained up to 50 bacteria per cell.

### 4.9. Host Defense Peptide Susceptibility Assay

Susceptibility of PB vs. RB isolates to host defense peptides (HDPs) in the presence or absence of relevant anti-staphylococcal antibiotics was determined by our established ultra-sensitive radial diffusion assay [85]. In brief, logarithmic-phase cells adjusted to 10^6^ CFU/mL were seeded into 10 mL of buffered 1% agarose. Piperazine-N,N′-bis(2-ethanesulfonic acid) (PIPES; Fisher Scientific, Waltham, MA, USA) or 2-(N-morpholino) ethanesulfonic acid (MES; Fisher Scientific, Waltham, MA, USA) buffer was used to adjust assay conditions to pH 7.5 or pH 5.5, respectively. These pH conditions were chosen to represent relevant anatomical contexts, namely, the bloodstream or acidic phagolysosome, respectively. Based on pilot studies, 10 μg of each HDP was added to wells in the seeded underlay matrix and incubated at 37 °C for 3 h. Wells containing vehicle (phosphate-buffered saline [PBS]) alone were included in each assay as an internal control. After 3 h of incubation, plates were overlaid with nutrient medium (trypticase soy agar; Fisher Scientific, Waltham, MA, USA) and incubated for 24 h at 37 °C. Zones of inhibition (ZOIs) were measured in diameter to the nearest millimeter. A minimum of two independent experiments were conducted on separate days for statistical analysis.

### 4.10. Mechanistic Fingerprinting

Six-parameter, multicolor flow cytometry was used to analyze four specific mechanisms of HDP action and two global effects associated with these mechanisms in PB or RB isolates: (i) perturbation of cell membrane (CM) energetics (ENR) (e.g., transmembrane potential), (ii) CM permeabilization (PRM), (iii) annexin V binding (ANX) (negatively charged phospholipids and CM turnover), (iv) caspase-like/metacaspase-like cell death protease induction (CSP), (v) osmodisruption (forward scatter [FSC]), and (vi) chromosomal condensation/cytoplasmic refractivity (side scatter [SSC] granularity) [37]. The following fluorophores were used with a FACSCalibur instrument (Becton Dickinson, Franklin Lakes, NJ, USA): 3,3-dipentyloxacarbocyanine (DiOC5) (excitation, 484 nm; emission, 660 nm) (Invitrogen, Carlsbad, CA, USA) for ENR, propidium iodide (PI) (excitation, 535 nm; emission, 620 nm) (Sigma, St. Louis, MO, USA) for PRM, annexin V-allophycocyanin conjugate (ANX-V) (excitation, 650 nm; emission, 660 nm) (Invitrogen, Carlsbad, CA, USA) for ANX, and CellEvent caspase-3/7 green (C-3/7) (excitation, 502 nm; emission, 530 nm) (Invitrogen, Carlsbad, CA, USA) for CSP. FSC and SSC were measured in parallel. Logarithmic-phase organisms were adjusted to 10^6^ CFU/mL in PIPES (pH 7.5) and exposed to 10 μg/mL of each HDP or 1 μg/mL of each antibiotic for 1 h at 37 °C. Based on extensive pilot data, these concentrations yielded approximately 50% survival, given the high inoculum of bacteria exposed. A triple-stain cocktail containing DiOC_5_ (0.5 μM), PI (5.0 μg/mL), and ANX-V (2.5 μL/mL) in 50 mM potassium-containing minimal essential medium (K^+^ MEM) (without phenol red; Sigma) was added to each sample following incubation. Samples were stained at room temperature for 15 min before flow cytometry. Parallel samples were incubated with 30 μL of C-3/7 reagent for 30 min at 37 °C following peptide exposure. After incubation, 400 mL of PBS was added to remove any background signal. Sodium dodecyl sulfate (SDS) (10%, wt/vol; Ambion) (a nonspecific perturbant of ENR and PRM) or buffers alone (K^+^ MEM or PBS) were controls in each experiment. Fluorescence of a minimum of 10,000 cells was acquired from each sample, and the results from a minimum of two independent studies conducted on different days were used for statistical analysis.

### 4.11. Statistical Analyses

Experimental results were compared by Student’s t-test or ANOVA with correction for multiple comparisons, as indicated in figure legends. Data were represented as mean ± standard deviation. *p*-values < 0.05 were considered statistically significant. Statistical analyses were implemented and graphs generated using the GraphPad Prism software (version 7.0; San Diego, CA, USA).

## Data Availability

The data are contained within the article (Figure 1, Figure 2, Figure 3, Figure 4, Figure 5, Figure 6, Figure 7 and Figure 9) and Supplementary Materials.

## Supplementary Materials

The following supporting information can be downloaded at https://www.mdpi.com/article/10.3390/antibiotics15040332/s1, Table S1: Significant values of Pearson correlation of PB vs. RB susceptibility to HDPs in the presence or absence of antibiotics. (A) pH 5.5; (B) pH 7.5. Relationships were determined using Pearson linear correlation analysis (n = 10). Data represent *p* values of Pearson correlation. Red values indicate statistically significant correlations (*p* < 0.5). Abbreviations as defined in text. Dashes in columns and rows indicate no correlation determinable due to lack of efficacy. Table S2: Statistically significant values of mechanistic fingerprints in PB and RB isolates due to HDPs and antibiotics. Data represent *p* values of t-tests (corrected for multiple comparison using Holm-Sidak) between PB vs. RB isolate for each condition and measurement. CNTL: untreated control; EtOH: ethanol; SDS: sodium dodecyl sulfate; HNP-1, human neutrophil peptide-1; HBD-2, human beta defensin-2; LL-37, cathelicidin; gRP-1, gamma RP-1; Dap, daptomycin; Van, vancomycin; FSC, forward scatter; SSC, side scatter; ENR, energetics; PRM, permeabilization; ANX, annexin V binding; CSP, caspase-like protease induction.

## Appendix A

Members of the MRSA Systems Immunobiology Group (MSIG) (in alphabetical order): Aaron S. Meyer, Alan J. Waring, Alexander Hoffmann, Arnold S. Bayer, Batu K. Sharma, David W. Gjertson, Elaine F. Reed, Felicia Ruffin, Felix M. Medie, Harry Picker-ing, Jackson L. Chin, Joshua T. Thaden, Jr., Kristina V. Bergersen, Liana C. Chan, Maura Rossetti, Rajesh Parmar, Richard Ahn, Scott G. Filler, Tsuyoshi Mikkaichi, Vance G. Fowler, Yan Q. Xiong and Yu-Ling Chang and Michael R. Yeaman.
