# Supplementary material for: Virulence Phenotypes Differentiate Persistent vs. Resolving Isolates of Human Staphylococcus aureus Bacteremia
Source: Antibiotics (Basel). 2026 Mar 25;15(4):332. doi: 10.3390/antibiotics15040332 (PMC13113033; doi:10.3390/antibiotics15040332)
Supplement: Supplementary file 1 [file antibiotics-15-00332-s001.zip › antibiotics-4112441-supplementary.pdf]

# Virulence phenotypes differentiate persistent vs. resolving isolates of human *Staphylococcus aureus* bacteremia

Supplemental Material

# Supplemental Table S1.

A

| pH 5.5 | PB Cef gRP-1 | PB Cef HBD-2 | PB Cef HNP-1 | PB Cef LL-37 | RB Cef gRP-1 | RB Cef HBD-2 | RB Cef HNP-1 | RB Cef LL-37 | PB Dap gRP-1 | PB Dap HBD-2 | PB Dap HNP-1 | PB Dap LL-37 | RB Dap gRP-1 | RB Dap HBD-2 | RB Dap HNP-1 | RB Dap LL-37 |
|---|---|---|---|---|---|---|---|---|---|---|---|---|---|---|---|---|
| PB Cef gRP-1 | | 0.029 | 0.144 | 0.035 | 0.080 | 0.053 | 0.176 | 0.308 | 0.554 | 0.283 | 0.722 | 0.277 | 0.585 | - | 0.787 | 0.801 |
| PB Cef HBD-2 | 0.029 | | 0.043 | 0.016 | 0.057 | 0.000 | 0.001 | 0.707 | 0.180 | 0.565 | 0.515 | 0.289 | 0.551 | - | 0.421 | 0.429 |
| PB Cef HNP-1 | 0.144 | 0.043 | | 0.155 | 0.301 | 0.067 | 0.073 | 0.752 | 0.937 | 0.014 | 0.884 | 0.414 | 0.547 | - | 0.682 | 0.682 |
| PB Cef LL-37 | 0.035 | 0.016 | 0.155 | | 0.003 | 0.005 | 0.007 | 0.155 | 0.225 | 0.526 | 0.260 | 0.091 | 0.039 | - | 0.336 | 0.358 |
| RB Cef gRP-1 | 0.080 | 0.057 | 0.301 | 0.003 | | 0.020 | 0.048 | 0.008 | 0.593 | 0.549 | 0.871 | 0.145 | 0.056 | - | 0.857 | 0.224 |
| RB Cef HBD-2 | 0.053 | <0.001 | 0.067 | 0.005 | 0.020 | | 0.004 | 0.463 | 0.369 | 0.422 | 0.555 | 0.334 | 0.398 | - | 0.254 | 0.605 |
| RB Cef HNP-1 | 0.176 | 0.001 | 0.073 | 0.007 | 0.048 | 0.004 | | 0.572 | 0.041 | 0.952 | 0.136 | 0.160 | 0.159 | - | 0.468 | 0.839 |
| RB Cef LL-37 | 0.308 | 0.707 | 0.752 | 0.155 | 0.008 | 0.463 | 0.572 | | 0.795 | 0.516 | 0.377 | 0.266 | 0.061 | - | 0.363 | 0.025 |
| PB Dap gRP-1 | 0.554 | 0.180 | 0.937 | 0.225 | 0.593 | 0.369 | 0.041 | 0.795 | | 0.076 | 0.007 | 0.024 | 0.640 | - | 0.330 | 0.660 |
| PB Dap HBD-2 | 0.283 | 0.565 | 0.014 | 0.526 | 0.549 | 0.422 | 0.952 | 0.516 | 0.076 | | 0.151 | 0.123 | 0.957 | - | 0.274 | 0.875 |
| PB Dap HNP-1 | 0.722 | 0.515 | 0.884 | 0.260 | 0.871 | 0.555 | 0.136 | 0.377 | 0.007 | 0.151 | | 0.241 | 0.522 | - | 0.110 | 0.793 |
| PB Dap LL-37 | 0.277 | 0.289 | 0.414 | 0.091 | 0.145 | 0.334 | 0.160 | 0.266 | 0.024 | 0.123 | 0.241 | | 0.222 | - | 0.392 | 0.360 |
| RB Dap gRP-1 | 0.585 | 0.551 | 0.547 | 0.039 | 0.056 | 0.398 | 0.159 | 0.061 | 0.640 | 0.957 | 0.522 | 0.222 | | - | 0.584 | 0.011 |
| RB Dap HBD-2 | - | - | - | - | - | - | - | - | - | - | - | - | - | | - | - |
| RB Dap HNP-1 | 0.787 | 0.421 | 0.682 | 0.336 | 0.857 | 0.254 | 0.468 | 0.363 | 0.330 | 0.274 | 0.110 | 0.392 | 0.584 | - | | 0.649 |
| RB Dap LL-37 | 0.801 | 0.429 | 0.682 | 0.358 | 0.224 | 0.605 | 0.839 | 0.025 | 0.660 | 0.875 | 0.793 | 0.360 | 0.011 | - | 0.649 | |
| PB Van gRP-1 | 0.383 | 0.823 | 0.538 | 0.297 | 0.667 | 0.953 | 0.666 | 0.882 | 0.376 | 0.619 | 0.188 | 0.835 | 0.987 | - | 0.792 | 0.705 |
| PB Van HBD-2 | - | - | - | - | - | - | - | - | - | - | - | - | - | - | - | - |
| PB Van HNP-1 | 0.411 | 0.783 | 0.567 | 0.270 | 0.581 | 0.507 | 0.226 | 0.887 | 0.537 | 0.781 | 0.081 | 0.902 | 0.029 | - | 0.190 | 0.488 |
| PB Van LL-37 | 0.787 | 0.942 | 0.714 | 0.937 | 0.957 | 0.764 | 0.819 | 0.839 | 0.865 | 0.619 | 0.882 | 0.844 | 0.212 | - | 0.603 | 0.530 |
| RB Van gRP-1 | 0.782 | 0.428 | 0.119 | 0.290 | 0.536 | 0.515 | 0.059 | 0.981 | 0.219 | 0.969 | 0.073 | 0.890 | 0.133 | - | 0.852 | 0.852 |
| RB Van HBD-2 | - | - | - | - | - | - | - | - | - | - | - | - | - | - | - | - |
| RB Van HNP-1 | 0.925 | 0.791 | 0.770 | 0.348 | 0.783 | 0.868 | 0.880 | 0.995 | 0.966 | 0.810 | 0.408 | 0.512 | 0.053 | - | 0.214 | 0.056 |
| RB Van LL-37 | 0.336 | 0.736 | 0.517 | 0.785 | 0.939 | 0.614 | 0.356 | 0.351 | 0.424 | 0.603 | 0.064 | 0.479 | 0.622 | - | 0.175 | 0.433 |
| PB No abx gRP-1 | 0.045 | 0.913 | 0.952 | 0.266 | 0.176 | 0.862 | 0.991 | 0.020 | 0.823 | 0.614 | 0.458 | 0.190 | 0.373 | - | 0.442 | 0.079 |
| PB No abx HBD-2 | - | - | - | - | - | - | - | - | - | - | - | - | - | - | - | - |
| PB No abx HNP-1 | 0.052 | 0.226 | 0.937 | 0.447 | 0.132 | 0.297 | 0.624 | 0.115 | 0.946 | 0.686 | 0.189 | 0.170 | 0.816 | - | 0.412 | 0.853 |
| PB No abx LL-37 | 0.757 | 0.714 | 0.886 | 0.650 | 0.425 | 0.787 | 0.888 | 0.214 | 0.913 | 0.758 | 0.775 | 0.929 | 0.997 | - | 0.633 | 0.810 |
| RB No abx gRP-1 | 0.069 | 0.513 | 0.051 | 0.117 | 0.129 | 0.321 | 0.715 | 0.088 | 0.229 | 0.002 | 0.261 | 0.699 | 0.199 | - | 0.557 | 0.155 |
| RB No abx HBD-2 | - | - | - | - | - | - | - | - | - | - | - | - | - | - | - | - |
| RB No abx HNP-1 | 0.369 | 0.713 | 0.836 | 0.145 | 0.159 | 0.405 | 0.754 | 0.430 | 0.647 | 0.790 | 0.874 | 0.732 | 0.064 | - | 0.085 | 0.142 |
| RB No abx LL-37 | 0.327 | 0.868 | 0.563 | 0.752 | 0.524 | 0.980 | 0.835 | 0.048 | 0.491 | 0.361 | 0.106 | 0.925 | 0.236 | - | 0.456 | 0.183 |

| pH 5.5 | PB Van gRP-1 | PB Van HBD-2 | PB Van HNP-1 | PB Van LL-37 | RB Van gRP-1 | RB Van HBD-2 | RB Van HNP-1 | RB Van LL-37 | PB Cntl gRP-1 | PB Cntl HBD-2 | PB Cntl HNP-1 | PB Cntl LL-37 | RB Cntl gRP-1 | RB Cntl HBD-2 | RB Cntl HNP-1 | RB Cntl LL-37 |
|---|---|---|---|---|---|---|---|---|---|---|---|---|---|---|---|---|
| PB Cef gRP-1 | 0.383 | - | 0.411 | 0.787 | 0.782 | - | 0.925 | 0.336 | 0.045 | - | 0.052 | 0.757 | 0.069 | - | 0.369 | 0.327 |
| PB Cef HBD-2 | 0.823 | - | 0.783 | 0.942 | 0.428 | - | 0.791 | 0.736 | 0.913 | - | 0.226 | 0.714 | 0.513 | - | 0.713 | 0.868 |
| PB Cef HNP-1 | 0.538 | - | 0.567 | 0.714 | 0.119 | - | 0.770 | 0.517 | 0.952 | - | 0.937 | 0.886 | 0.051 | - | 0.836 | 0.563 |
| PB Cef LL-37 | 0.297 | - | 0.270 | 0.937 | 0.290 | - | 0.348 | 0.785 | 0.266 | - | 0.447 | 0.650 | 0.117 | - | 0.145 | 0.752 |
| RB Cef gRP-1 | 0.667 | - | 0.581 | 0.957 | 0.536 | - | 0.783 | 0.939 | 0.176 | - | 0.132 | 0.425 | 0.129 | - | 0.159 | 0.524 |
| RB Cef HBD-2 | 0.953 | - | 0.507 | 0.764 | 0.515 | - | 0.868 | 0.614 | 0.862 | - | 0.297 | 0.787 | 0.321 | - | 0.405 | 0.980 |
| RB Cef HNP-1 | 0.666 | - | 0.226 | 0.819 | 0.059 | - | 0.880 | 0.356 | 0.991 | - | 0.624 | 0.888 | 0.715 | - | 0.754 | 0.835 |
| RB Cef LL-37 | 0.882 | - | 0.887 | 0.839 | 0.981 | - | 0.995 | 0.351 | 0.020 | - | 0.115 | 0.214 | 0.088 | - | 0.430 | 0.048 |
| PB Dap gRP-1 | 0.376 | - | 0.537 | 0.865 | 0.219 | - | 0.966 | 0.424 | 0.823 | - | 0.946 | 0.913 | 0.229 | - | 0.647 | 0.491 |
| PB Dap HBD-2 | 0.619 | - | 0.781 | 0.619 | 0.969 | - | 0.810 | 0.603 | 0.614 | - | 0.686 | 0.758 | 0.002 | - | 0.790 | 0.361 |
| PB Dap HNP-1 | 0.188 | - | 0.081 | 0.882 | 0.073 | - | 0.408 | 0.064 | 0.458 | - | 0.189 | 0.775 | 0.261 | - | 0.874 | 0.106 |
| PB Dap LL-37 | 0.835 | - | 0.902 | 0.844 | 0.890 | - | 0.512 | 0.479 | 0.190 | - | 0.170 | 0.929 | 0.699 | - | 0.732 | 0.925 |
| RB Dap gRP-1 | 0.987 | - | 0.029 | 0.212 | 0.133 | - | 0.053 | 0.622 | 0.373 | - | 0.816 | 0.997 | 0.199 | - | 0.064 | 0.236 |
| RB Dap HBD-2 | - | - | - | - | - | - | - | - | - | - | - | - | - | - | - | - |
| RB Dap HNP-1 | 0.792 | - | 0.190 | 0.603 | 0.852 | - | 0.214 | 0.175 | 0.442 | - | 0.412 | 0.633 | 0.557 | - | 0.085 | 0.456 |
| RB Dap LL-37 | 0.705 | - | 0.488 | 0.530 | 0.852 | - | 0.056 | 0.433 | 0.079 | - | 0.853 | 0.810 | 0.155 | - | 0.142 | 0.183 |
| PB Van gRP-1 | | - | 0.880 | 0.548 | 0.424 | - | 0.637 | 0.873 | 0.405 | - | 0.824 | 0.564 | 0.502 | - | 0.877 | 0.396 |
| PB Van HBD-2 | - | | - | - | - | - | - | - | - | - | - | - | - | - | - | - |
| PB Van HNP-1 | 0.880 | - | | 0.371 | 0.011 | - | 0.195 | 0.012 | 0.386 | - | 0.037 | 0.704 | 0.894 | - | 0.259 | 0.984 |
| PB Van LL-37 | 0.548 | - | 0.371 | | 0.268 | - | 0.487 | 0.293 | 0.796 | - | 0.394 | 0.302 | 0.844 | - | 0.212 | 0.182 |
| RB Van gRP-1 | 0.424 | - | 0.011 | 0.268 | | - | 0.690 | 0.011 | 0.552 | - | 0.106 | 0.802 | 0.873 | - | 0.822 | 0.896 |
| RB Van HBD-2 | - | - | - | - | - | | - | - | - | - | - | - | - | - | - | - |
| RB Van HNP-1 | 0.637 | - | 0.195 | 0.487 | 0.690 | - | | 0.915 | 0.971 | - | 0.379 | 0.194 | 0.529 | - | 0.029 | 0.846 |
| RB Van LL-37 | 0.873 | - | 0.012 | 0.293 | 0.011 | - | 0.915 | | 0.097 | - | 0.013 | 0.691 | 0.490 | - | 0.567 | 0.434 |
| PB No abx gRP-1 | 0.405 | - | 0.386 | 0.796 | 0.552 | - | 0.971 | 0.097 | | - | 0.093 | 0.392 | 0.098 | - | 0.636 | 0.033 |
| PB No abx HBD-2 | - | - | - | - | - | - | - | - | - | | - | - | - | - | - | - |
| PB No abx HNP-1 | 0.824 | - | 0.037 | 0.394 | 0.106 | - | 0.379 | 0.013 | 0.093 | - | | 0.961 | 0.510 | - | 0.769 | 0.522 |
| PB No abx LL-37 | 0.564 | - | 0.704 | 0.302 | 0.802 | - | 0.194 | 0.691 | 0.392 | - | 0.961 | | 0.722 | - | 0.688 | 0.627 |
| RB No abx gRP-1 | 0.502 | - | 0.894 | 0.844 | 0.873 | - | 0.529 | 0.490 | 0.098 | - | 0.510 | 0.722 | | - | 0.186 | 0.081 |
| RB No abx HBD-2 | - | - | - | - | - | - | - | - | - | - | - | - | - | | - | - |
| RB No abx HNP-1 | 0.877 | - | 0.259 | 0.212 | 0.822 | - | 0.029 | 0.567 | 0.636 | - | 0.769 | 0.688 | 0.186 | - | | 0.663 |
| RB No abx LL-37 | 0.396 | - | 0.984 | 0.182 | 0.896 | - | 0.846 | 0.434 | 0.033 | - | 0.522 | 0.627 | 0.081 | - | 0.663 | |

B

| pH 7.5 | PB Cef gRP-1 | PB Cef HBD-2 | PB Cef HNP-1 | PB Cef LL-37 | RB Cef gRP-1 | RB Cef HBD-2 | RB Cef HNP-1 | RB Cef LL-37 | PB Dap gRP-1 | PB Dap HBD-2 | PB Dap HNP-1 | PB Dap LL-37 | RB Dap gRP-1 | RB Dap HBD-2 | RB Dap HNP-1 | RB Dap LL-37 |
|---|---|---|---|---|---|---|---|---|---|---|---|---|---|---|---|---|
| PB Cef gRP-1 | | 0.228 | 0.255 | <0.001 | 0.007 | 0.050 | 0.201 | 0.026 | 0.001 | 0.052 | 0.005 | 0.002 | 0.049 | <0.001 | 0.067 | 0.011 |
| PB Cef HBD-2 | 0.228 | | 0.001 | 0.286 | 0.387 | 0.250 | 0.039 | 0.777 | 0.916 | 0.612 | 0.460 | 0.892 | 0.631 | 0.452 | 0.901 | 0.499 |
| PB Cef HNP-1 | 0.255 | 0.001 | | 0.184 | 0.258 | 0.069 | 0.010 | 0.915 | 0.963 | 0.692 | 0.621 | 0.583 | 0.425 | 0.450 | 0.704 | 0.279 |
| PB Cef LL-37 | <0.001 | 0.286 | 0.184 | | 0.004 | 0.019 | 0.158 | 0.042 | 0.002 | 0.043 | 0.016 | <0.001 | 0.080 | <0.001 | 0.084 | 0.007 |
| RB Cef gRP-1 | 0.007 | 0.387 | 0.258 | 0.004 | | 0.001 | 0.017 | 0.002 | 0.016 | 0.008 | 0.001 | 0.005 | 0.002 | 0.002 | 0.006 | <0.001 |
| RB Cef HBD-2 | 0.050 | 0.250 | 0.069 | 0.019 | 0.001 | | 0.008 | 0.043 | 0.074 | 0.064 | 0.052 | 0.019 | 0.007 | 0.027 | 0.010 | 0.002 |
| RB Cef HNP-1 | 0.201 | 0.039 | 0.010 | 0.158 | 0.017 | 0.008 | | 0.277 | 0.642 | 0.420 | 0.191 | 0.399 | 0.032 | 0.145 | 0.067 | 0.015 |
| RB Cef LL-37 | 0.026 | 0.777 | 0.915 | 0.042 | 0.002 | 0.043 | 0.277 | | 0.005 | 0.001 | 0.001 | 0.014 | 0.024 | 0.011 | 0.023 | 0.014 |
| PB Dap gRP-1 | 0.001 | 0.916 | 0.963 | 0.002 | 0.016 | 0.074 | 0.642 | 0.005 | | 0.003 | 0.009 | <0.001 | 0.049 | 0.001 | 0.034 | 0.017 |
| PB Dap HBD-2 | 0.052 | 0.612 | 0.692 | 0.043 | 0.008 | 0.064 | 0.420 | 0.001 | 0.003 | | 0.029 | 0.008 | 0.031 | 0.010 | 0.004 | 0.004 |
| PB Dap HNP-1 | 0.005 | 0.460 | 0.621 | 0.016 | 0.001 | 0.052 | 0.191 | 0.001 | 0.009 | 0.029 | | 0.012 | 0.017 | 0.002 | 0.050 | 0.025 |
| PB Dap LL-37 | 0.002 | 0.892 | 0.583 | <0.001 | 0.005 | 0.019 | 0.399 | 0.014 | <0.001 | 0.008 | 0.012 | | 0.057 | 0.001 | 0.054 | 0.011 |
| RB Dap gRP-1 | 0.049 | 0.631 | 0.425 | 0.080 | 0.002 | 0.007 | 0.032 | 0.024 | 0.049 | 0.031 | 0.017 | 0.057 | | 0.021 | <0.001 | 0.001 |
| RB Dap HBD-2 | 0.000 | 0.452 | 0.450 | 0.000 | 0.002 | 0.027 | 0.145 | 0.011 | 0.001 | 0.010 | 0.002 | 0.001 | 0.021 | | 0.015 | 0.002 |
| RB Dap HNP-1 | 0.067 | 0.901 | 0.704 | 0.084 | 0.006 | 0.010 | 0.067 | 0.023 | 0.034 | 0.004 | 0.050 | 0.054 | <0.001 | 0.015 | | <0.001 |
| RB Dap LL-37 | 0.011 | 0.499 | 0.279 | 0.007 | <0.001 | 0.002 | 0.015 | 0.014 | 0.017 | 0.004 | 0.025 | 0.011 | 0.001 | 0.002 | <0.001 | |
| PB Van gRP-1 | 0.074 | 0.337 | 0.625 | 0.071 | 0.059 | 0.212 | 0.835 | 0.025 | 0.001 | 0.008 | 0.056 | 0.008 | 0.068 | 0.030 | 0.059 | 0.046 |
| PB Van HBD-2 | 0.272 | 0.970 | 0.833 | 0.506 | 0.092 | 0.516 | 0.225 | 0.083 | 0.313 | 0.080 | 0.118 | 0.554 | 0.028 | 0.187 | 0.033 | 0.054 |
| PB Van HNP-1 | 0.103 | 0.999 | 0.532 | 0.061 | 0.001 | 0.002 | 0.061 | 0.012 | 0.027 | 0.011 | 0.023 | 0.015 | 0.002 | 0.024 | 0.003 | 0.001 |
| PB Van LL-37 | 0.085 | 0.798 | 0.583 | 0.047 | 0.058 | 0.114 | 0.552 | 0.105 | 0.016 | 0.132 | 0.101 | 0.008 | 0.128 | 0.093 | 0.254 | 0.091 |
| RB Van gRP-1 | 0.002 | 0.734 | 0.748 | 0.001 | 0.003 | 0.030 | 0.345 | 0.001 | <0.001 | 0.002 | 0.001 | <0.001 | 0.055 | 0.000 | 0.039 | 0.011 |
| RB Van HBD-2 | 0.124 | 0.365 | 0.275 | 0.086 | 0.002 | 0.030 | 0.007 | 0.024 | 0.195 | 0.040 | 0.045 | 0.144 | 0.048 | 0.038 | 0.045 | 0.004 |
| RB Van HNP-1 | 0.209 | 0.534 | 0.307 | 0.109 | 0.020 | 0.002 | 0.019 | 0.179 | 0.245 | 0.081 | 0.179 | 0.117 | 0.029 | 0.056 | 0.008 | 0.010 |
| RB Van LL-37 | 0.003 | 0.547 | 0.694 | 0.003 | 0.023 | 0.052 | 0.527 | 0.004 | 0.001 | 0.015 | 0.006 | 0.001 | 0.163 | 0.002 | 0.113 | 0.057 |
| PB No abx gRP-1 | 0.006 | 0.789 | 0.604 | 0.001 | 0.000 | 0.009 | 0.120 | 0.002 | 0.002 | <0.001 | 0.006 | 0.001 | 0.022 | <0.001 | 0.009 | <0.001 |
| PB No abx HBD-2 | 0.141 | 0.854 | 0.927 | 0.272 | 0.084 | 0.080 | 0.312 | 0.038 | 0.077 | 0.043 | 0.047 | 0.187 | 0.008 | 0.052 | 0.002 | 0.051 |
| PB No abx HNP-1 | 0.027 | 0.202 | 0.361 | 0.049 | 0.003 | 0.076 | 0.033 | 0.004 | 0.090 | 0.030 | 0.008 | 0.128 | 0.047 | 0.011 | 0.047 | 0.007 |
| PB No abx LL-37 | 0.183 | 0.990 | 0.646 | 0.056 | 0.026 | 0.105 | 0.317 | 0.051 | 0.084 | 0.055 | 0.094 | 0.026 | 0.409 | 0.083 | 0.387 | 0.090 |
| RB No abx gRP-1 | 0.008 | 0.555 | 0.491 | 0.010 | <0.001 | 0.008 | 0.043 | 0.001 | 0.007 | 0.001 | 0.004 | 0.011 | 0.003 | 0.001 | 0.001 | <0.001 |
| RB No abx HBD-2 | 0.060 | 0.796 | 0.406 | 0.016 | <0.001 | 0.007 | 0.059 | 0.012 | 0.035 | 0.012 | 0.019 | 0.006 | 0.037 | 0.017 | 0.047 | 0.003 |
| RB No abx HNP-1 | 0.657 | 0.707 | 0.878 | 0.447 | 0.178 | 0.228 | 0.239 | 0.232 | 0.444 | 0.023 | 0.702 | 0.426 | 0.305 | 0.300 | 0.064 | 0.051 |
| RB No abx LL-37 | 0.228 | 0.629 | 0.915 | 0.447 | 0.110 | 0.298 | 0.180 | 0.059 | 0.299 | 0.052 | 0.115 | 0.564 | 0.035 | 0.113 | 0.010 | 0.050 |

| pH 7.5 | PB Van gRP-1 | PB Van HBD-2 | PB Van HNP-1 | PB Van LL-37 | RB Van gRP-1 | RB Van HBD-2 | RB Van HNP-1 | RB Van LL-37 | PB Cntl gRP-1 | PB Cntl HBD-2 | PB Cntl HNP-1 | PB Cntl LL-37 | RB Cntl gRP-1 | RB Cntl HBD-2 | RB Cntl HNP-1 | RB Cntl LL-37 |
|---|---|---|---|---|---|---|---|---|---|---|---|---|---|---|---|---|
| PB Cef gRP-1 | 0.074 | 0.272 | 0.103 | 0.085 | 0.002 | 0.124 | 0.209 | 0.003 | 0.006 | 0.141 | 0.027 | 0.183 | 0.008 | 0.060 | 0.657 | 0.228 |
| PB Cef HBD-2 | 0.337 | 0.970 | 0.999 | 0.798 | 0.734 | 0.365 | 0.534 | 0.547 | 0.789 | 0.854 | 0.202 | 0.990 | 0.555 | 0.796 | 0.707 | 0.629 |
| PB Cef HNP-1 | 0.625 | 0.833 | 0.532 | 0.583 | 0.748 | 0.275 | 0.307 | 0.694 | 0.604 | 0.927 | 0.361 | 0.646 | 0.491 | 0.406 | 0.878 | 0.915 |
| PB Cef LL-37 | 0.071 | 0.506 | 0.061 | 0.047 | 0.001 | 0.086 | 0.109 | 0.003 | 0.001 | 0.272 | 0.049 | 0.056 | 0.010 | 0.016 | 0.447 | 0.447 |
| RB Cef gRP-1 | 0.059 | 0.092 | 0.001 | 0.058 | 0.003 | 0.002 | 0.020 | 0.023 | <0.001 | 0.084 | 0.003 | 0.026 | <0.001 | <0.001 | 0.178 | 0.110 |
| RB Cef HBD-2 | 0.212 | 0.516 | 0.002 | 0.114 | 0.030 | 0.030 | 0.002 | 0.052 | 0.009 | 0.080 | 0.076 | 0.105 | 0.008 | 0.007 | 0.228 | 0.298 |
| RB Cef HNP-1 | 0.835 | 0.225 | 0.061 | 0.552 | 0.345 | 0.007 | 0.019 | 0.527 | 0.120 | 0.312 | 0.033 | 0.317 | 0.043 | 0.059 | 0.239 | 0.180 |
| RB Cef LL-37 | 0.025 | 0.083 | 0.012 | 0.105 | 0.001 | 0.024 | 0.179 | 0.004 | 0.002 | 0.038 | 0.004 | 0.051 | 0.001 | 0.012 | 0.232 | 0.059 |
| PB Dap gRP-1 | 0.001 | 0.313 | 0.027 | 0.016 | <0.001 | 0.195 | 0.245 | 0.001 | 0.002 | 0.077 | 0.090 | 0.084 | 0.007 | 0.035 | 0.444 | 0.299 |
| PB Dap HBD-2 | 0.008 | 0.080 | 0.011 | 0.132 | 0.002 | 0.040 | 0.081 | 0.015 | <0.001 | 0.043 | 0.030 | 0.055 | 0.001 | 0.012 | 0.023 | 0.052 |
| PB Dap HNP-1 | 0.056 | 0.118 | 0.023 | 0.101 | 0.001 | 0.045 | 0.179 | 0.006 | 0.006 | 0.047 | 0.008 | 0.094 | 0.004 | 0.019 | 0.702 | 0.115 |
| PB Dap LL-37 | 0.008 | 0.554 | 0.015 | 0.008 | <0.001 | 0.144 | 0.117 | 0.001 | 0.001 | 0.187 | 0.128 | 0.026 | 0.011 | 0.006 | 0.426 | 0.564 |
| RB Dap gRP-1 | 0.068 | 0.028 | 0.002 | 0.128 | 0.055 | 0.048 | 0.029 | 0.163 | 0.022 | 0.008 | 0.047 | 0.409 | 0.003 | 0.037 | 0.305 | 0.035 |
| RB Dap HBD-2 | 0.030 | 0.187 | 0.024 | 0.093 | <0.001 | 0.038 | 0.056 | 0.002 | 0.000 | 0.052 | 0.011 | 0.083 | 0.001 | 0.017 | 0.300 | 0.113 |
| RB Dap HNP-1 | 0.059 | 0.033 | 0.003 | 0.254 | 0.039 | 0.045 | 0.008 | 0.113 | 0.009 | 0.002 | 0.047 | 0.387 | 0.001 | 0.047 | 0.064 | 0.010 |
| RB Dap LL-37 | 0.046 | 0.054 | 0.001 | 0.091 | 0.011 | 0.004 | 0.010 | 0.057 | 0.000 | 0.051 | 0.007 | 0.090 | <0.001 | 0.003 | 0.051 | 0.050 |
| PB Van gRP-1 | | 0.214 | 0.011 | 0.002 | 0.010 | 0.174 | 0.476 | 0.066 | 0.021 | 0.194 | 0.180 | 0.056 | 0.022 | 0.035 | 0.415 | 0.486 |
| PB Van HBD-2 | 0.214 | | 0.189 | 0.598 | 0.347 | 0.064 | 0.534 | 0.694 | 0.165 | 0.148 | 0.025 | 0.708 | 0.038 | 0.244 | 0.193 | 0.004 |
| PB Van HNP-1 | 0.011 | 0.189 | | 0.018 | 0.013 | 0.011 | 0.018 | 0.085 | 0.004 | 0.060 | 0.054 | 0.029 | 0.002 | 0.001 | 0.161 | 0.243 |
| PB Van LL-37 | 0.002 | 0.598 | 0.018 | | 0.050 | 0.213 | 0.629 | 0.159 | 0.062 | 0.715 | 0.340 | 0.028 | 0.100 | 0.018 | 0.915 | 0.753 |
| RB Van gRP-1 | 0.010 | 0.347 | 0.013 | 0.050 | | 0.051 | 0.101 | <0.001 | <0.001 | 0.058 | 0.018 | 0.021 | 0.001 | 0.009 | 0.318 | 0.210 |
| RB Van HBD-2 | 0.174 | 0.064 | 0.011 | 0.213 | 0.051 | | 0.057 | 0.187 | 0.008 | 0.299 | <0.001 | 0.014 | 0.001 | 0.002 | 0.041 | 0.099 |
| RB Van HNP-1 | 0.476 | 0.534 | 0.018 | 0.629 | 0.101 | 0.057 | | 0.148 | 0.024 | 0.043 | 0.161 | 0.252 | 0.027 | 0.047 | 0.057 | 0.142 |
| RB Van LL-37 | 0.066 | 0.694 | 0.085 | 0.159 | <0.001 | 0.187 | 0.148 | | 0.005 | 0.067 | 0.059 | 0.081 | 0.016 | 0.066 | 0.489 | 0.289 |
| PB No abx gRP-1 | 0.021 | 0.165 | 0.004 | 0.062 | <0.001 | 0.008 | 0.024 | 0.005 | | 0.107 | 0.010 | 0.009 | <0.001 | <0.001 | 0.057 | 0.138 |
| PB No abx HBD-2 | 0.194 | 0.148 | 0.060 | 0.715 | 0.058 | 0.299 | 0.043 | 0.067 | 0.107 | | 0.132 | 0.910 | 0.026 | 0.370 | 0.365 | 0.007 |
| PB No abx HNP-1 | 0.180 | 0.025 | 0.054 | 0.340 | 0.018 | <0.001 | 0.161 | 0.059 | 0.010 | 0.132 | | 0.080 | 0.001 | 0.024 | 0.129 | 0.019 |
| PB No abx LL-37 | 0.056 | 0.708 | 0.029 | 0.028 | 0.021 | 0.014 | 0.252 | 0.081 | 0.009 | 0.910 | 0.080 | | 0.049 | <0.001 | 0.186 | 0.975 |
| RB No abx gRP-1 | 0.022 | 0.038 | 0.002 | 0.100 | 0.001 | 0.001 | 0.027 | 0.016 | <0.001 | 0.026 | 0.001 | 0.049 | | 0.003 | 0.062 | 0.023 |
| RB No abx HBD-2 | 0.035 | 0.244 | 0.001 | 0.018 | 0.009 | 0.002 | 0.047 | 0.066 | <0.001 | 0.370 | 0.024 | <0.001 | 0.003 | | 0.110 | 0.430 |
| RB No abx HNP-1 | 0.415 | 0.193 | 0.161 | 0.915 | 0.318 | 0.041 | 0.057 | 0.489 | 0.057 | 0.365 | 0.129 | 0.186 | 0.062 | 0.110 | | 0.088 |
| RB No abx LL-37 | 0.486 | 0.004 | 0.243 | 0.753 | 0.210 | 0.099 | 0.142 | 0.289 | 0.138 | 0.007 | 0.019 | 0.975 | 0.023 | 0.430 | 0.088 | |

**Supplemental Table S1. Significant values of Pearson correlation of PB vs. RB susceptibility to HDPs in the presence or absence of antibiotics.** (A) pH 5.5; (B) pH 7.5. Relationships were determined using Pearson linear correlation analysis (n = 10). Data represent P values of Pearson correlation. Red values indicate statistically significant correlations (P < 0.5). Abbreviations as defined in text. Dashes in columns and rows indicate no correlation determinable due to lack of efficacy.

# Supplemental Table S2.

| | FSC | SSC | ENR | PRM | ANX | CSP |
|---|---|---|---|---|---|---|
| HNP-1 | 0.269 | 0.718 | 0.078 | 0.788 | 0.050 | 0.002 |
| HBD-2 | 0.285 | 0.392 | 0.028 | 0.500 | 0.970 | 0.048 |
| LL-37 | 0.745 | 0.522 | 0.017 | 0.281 | 0.516 | 0.707 |
| gRP-1 | 0.160 | 0.398 | 0.060 | 0.396 | 0.546 | 0.442 |
| Dap | 0.224 | 0.096 | 0.032 | 0.580 | 0.106 | 0.840 |
| Van | 0.095 | 0.202 | 0.286 | 0.489 | 0.005 | 0.010 |

Supplemental Table S2. Statistically significant values of mechanistic fingerprints in PB and RB isolates due to HDPs and antibiotics. Data represent P values of t-tests (corrected for multiple comparison using Holm-Sidak) between PB vs. RB isolate for each condition and measurement. CNTL: untreated control; EtOH: ethanol; SDS: sodium dodecyl sulfate; HNP-1, human neutrophil peptide-1; HBD-2, human beta defensin-2; LL-37, cathelicidin; gRP-1, gamma RP-1; Dap, daptomycin; Van, vancomycin; FSC, forward scatter; SSC, side scatter; ENR, energetics; PRM, permeabilization; ANX, annexin V binding; CSP, caspase-like protease induction.
